# COVID-19: is it just a lung disease? A case-based review

**DOI:** 10.1007/s42399-020-00418-6

**Published:** 2020-07-28

**Authors:** Valerio Spuntarelli, M. Luciani, E. Bentivegna, V. Marini, F. Falangone, G. Conforti, E. S. Rachele, P. Martelletti

**Affiliations:** Internal Medicine Ward, Sant’Andrea Hospital – La Sapienza University, Via di Grottarossa 1035, Rome, Italy

**Keywords:** “COVID-19” “OR” “SARS” “OR” “SARS – COV 2” “AND” “systemic disease”, “nephropathy”, “cardiac pathology”, “central nervous system”

## Abstract

Due to its extreme virulence, COVID-19 virus has rapidly spread, developing a severe pandemic. SARS-COV-2 mostly affected the respiratory tract, causing a severe acute lung failure. Although the infection of airways, COVID-19 can be associated with chronic and systemic damages still not so much known. The purpose of this research is to collect recent evidence in literature about systemic diseases caused by COVID-19. The format of the present article has features of a systematic case-based review (level of evidence), and it is structured as a case series report (patients of our COVID-19 Medicine Ward have been selected as cases). Data for this review have been selected systematically, taking evidence only from indexed journals and databases: PubMed, Scopus, MEDLINE, and Cochrane systems. Papers chosen included systematic reviews, case series, clinical cases, meta-analysis studies, and RCTs. We start collecting studies since 2003. The main keywords used were “COVID-19” “OR” “SARS” “OR” “SARS – COV 2” “AND” “systemic disease” / “nephropathy” / “cardiac pathology” / “central nervous system.” Clinical cases belong to our COVID-19 Medicine Ward. One of the most severe COVID-19 clinical presentations includes cardiovascular problems, like myocarditis, pericarditis, and acute hearth failure. Cytokine release syndrome caused by COVID-19 develops severe acute kidney failure. It is still unknown the way coronavirus damages the liver, brain, and reproductive system. Considering the majority of the new studies about this pathology, it issues that COVID-19 is considered to be a multi-organ disease.

## Introduction

COVID-19 pandemic reached 3.78 million confirmed reported cases worldwide, and it is generally associated to the acronym that precedes its name: severe acute respiratory syndrome (SARS). However, the bottom of the iceberg is being progressively unveiled since it is far more than simply a severe interstitial pneumonia. There is a gap in knowledge of pathophysiological process that allows COVID-19 to be considered a multi-organ disease in all respects.

We looked for main papers about SARS and COVID-19 as systemic diseases, focusing on cardiovascular, renal, liver, nervous, and reproductive systems.

## Cardiovascular System

The SARS-COV-2 virus not only causes viral pneumonia; however, it also has major implications for the cardiovascular system, but the extent, severity, and duration are still to be defined. In a study of 75 patients, two patients had succumbed to acute myocardial infarction [1]**.**

A study from Huang et al. demonstrated that myocardial injury, defined by an increase in hs-cTnI levels (> 28 pg/mL), occurred on 5 out of 41 COVID-19 patients. Noteworthy is the fact that 80% of these patients required ICU management demonstrating that myocardial damage in SARS-COV-2 infection is severe [2]**.**

A prospective study investigating left ventricular performance in 46 patients with severe acute respiratory syndrome showed subclinical diastolic impairment without systolic involvement [3]**.** Another study in 121 patients with SARS identified cardiovascular complications including tachycardia (72%), hypotension (50%), bradycardia (15%), transient cardiomegaly (11%), and transient paroxysmal atrial fibrillation in only 1 patient, although usually self-limiting [4]**.** A study from Singapore reported postmortem examinations: the presence of pulmonary embolism (PE) and deep vein thrombosis and acute myocardial infarction is of great clinical interest, but the generalizability of this limited study is not established [5]**.**

Anyway, it is well recognized that SARS-COV-2 infection alters coagulation pathway. Zhou et al. demonstrated that non-survivor COVID-19 patients show significant higher levels of plasma D-dimer, activated partial thromboplastin times, and prothrombin times compared with survivors [6].

Pathological findings of COVID-19 associated with acute respiratory distress syndrome showed few interstitial mononuclear inflammatory infiltrates, but no other substantial damage in the heart tissue [7]**.** Shi S. et al [8] reported that between 57 out of 416 patients, 10.6% had coronary heart disease, 4.1% had heart failure, and 5.3% had cerebrovascular disease underlying the importance of cardiac injury in COVID-19. A case report highlights myocarditis as a complication associated with COVID-19, even without symptoms and signs of interstitial pneumonia in an otherwise healthy 53-year-old white woman [8]**.** Another case report [9] of an infected 69-year-old man, from Lombardy, Italy, concluded that COVID-19 infection was the most likely cause of myocarditis since tests for common causes of myocarditis (parvovirus B19, human herpes virus, Epstein-Barr virus, enterovirus, cytomegalovirus, adenovirus, HIV, and hepatitis C virus) were negative. Hydrocortisone improved its clinical picture. Also, very recently, an association between COVID-19 and medium size vasculitis—Kawasaki disease—has emerged. According to older studies, a suspected association between HCoVs and Kawasaki disease could not be confirmed. However, Shirato K. et al [10] postulated that HCoV-229E is a possible causative agent for Kawasaki disease. Future case control studies are needed to settle the issue.

### Clinical Case

**Fig. 1:**
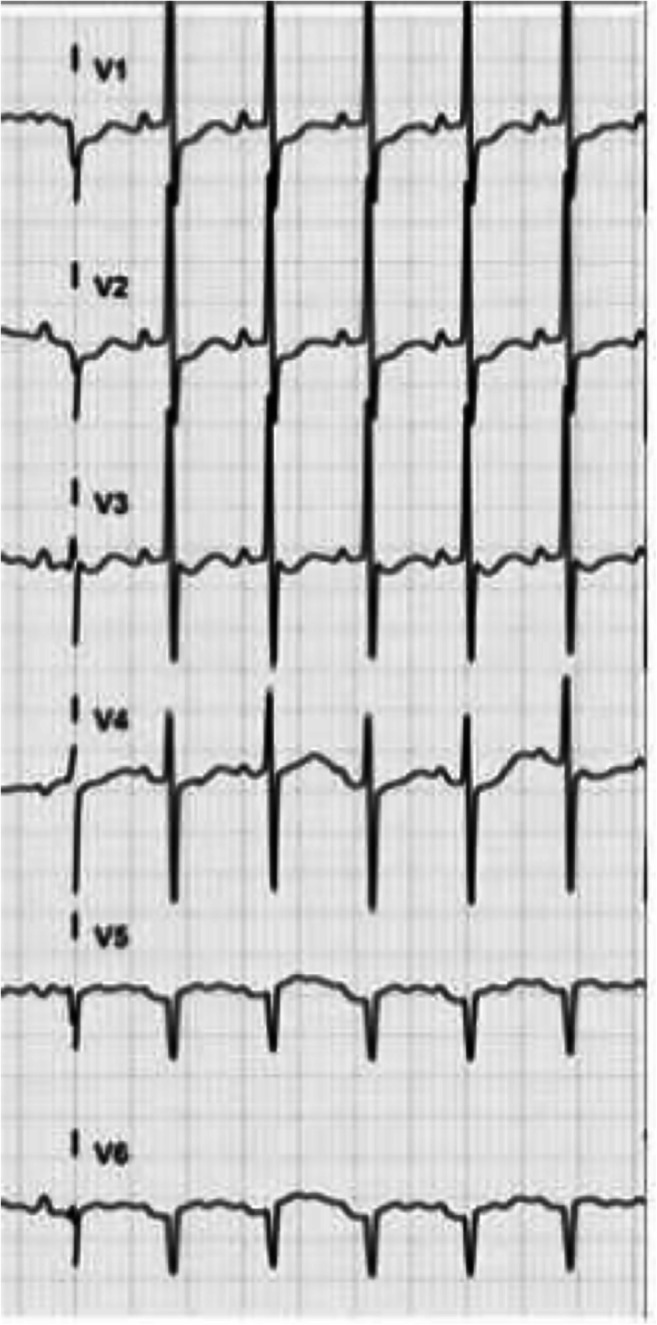
Moderate ST depression in most precordial leads

A 56-year-old male arrived to emergency for chest pain, tachycardia, and dyspnea. Blood gas analysis showed mild respiratory failure. SARS-COV-2 buffer was positive. Blood analysis pointed out an increase of serum myoglobin, high sensitive troponine, and MB creatine kinase. Electrocardiogram was typical for myocarditis (Fig.1).

## Kidney

Three potential pathogenic mechanisms of kidney injury secondary to COVID-19 have been identified. First, cytokine release syndrome (CRS)—aka cytokine storm—has been implicated in the development of intrarenal inflammation and increased vascular permeability. IL-6, markedly elevated in ARDS patients, plays a major role in the pathogenesis of the disease. In CRS patients who have undergone CAR-T cell therapy, the cytokine is the target of the therapy with tocilizumab and, thus, is now also being used empirically in patients with COVID-19. Second, alveolar-tubular and cardiorenal crosstalk has been implicated. Acute kidney injury (AKI) is the most frequent extra-pulmonary organ failure in acute respiratory distress syndrome (ARDS), according to a recently confirmed bidirectional organ crosstalk, possibly IL-6 mediated. IL-6 is not a risk factor for ARDS development, but high levels are associated to increased mortality rate in patients with ARDS. Independent risk factors are older age, disease severity, diabetes mellitus, and acidosis. Finally renin-angiotensin-aldosterone system increases both glomerular capillary and systemic hypertension, inducing hemodynamic injury to the vascular endothelium and glomerulus. Angiotensin II and aldosterone may also promote kidney damage with direct profibrotic and proinflammatory actions.

As shown in the clinical case below, the further renal impairment caused by COVID, in already nephropathic patients, can be considered a mortality predictor.

### Clinical Case

**Fig. 2:**
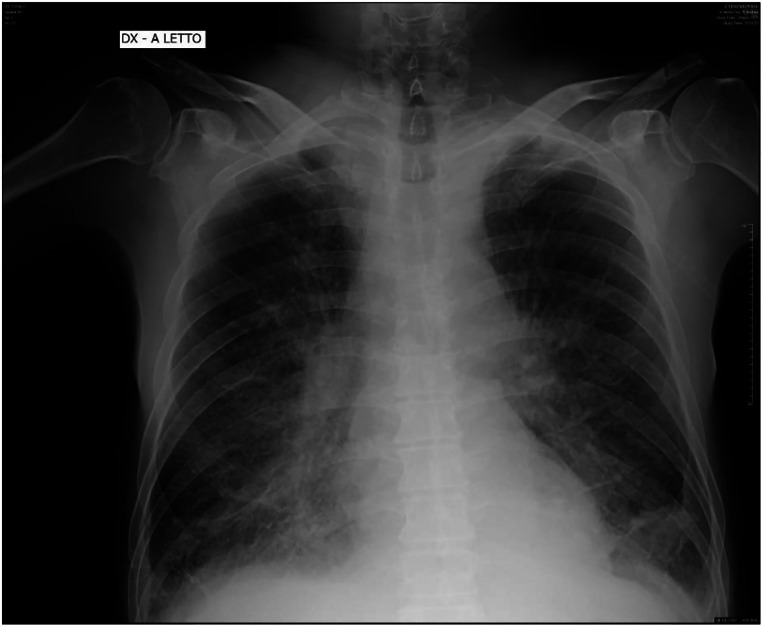
Emergency chest X-ray with severe interstitial edema

A 62-year-old woman with chronic kidney failure was hospitalized in emergency ward for metabolic acidosis. SARS-COV-2 buffer was positive. Blood exams showed an important increase of serum creatinine. During observation, patient developed an important ARDS with lung edema (Fig. 2). She died after 2 days of noninvasive ventilation.

## Liver

It is less clear whether COVID-19 damages the liver. Case reports have described increased levels of alanine aminotransferase (ALT), aspartate aminotransferase (AST), and bilirubin, indicating liver involvement, in which frequency is related to the severity of COVID-19 illness. The same was observed during previous SARS and MERS epidemics. On the other hand, Mansoor N.B. et al [11] reassure about the relationship, describing that the previous studies on the issue actually suggest that clinically significant liver injury is uncommon, even when data for the most severely ill patients are selected. Liver injury can also alternatively due to hypoxemia, dysregulated immune response, or drug toxicity.

## Nervous System

One-third of patients affected with COVID-19 have nervous system involvement. Virus can be detected in brain specimens and in cerebrospinal fluid. Acute onset smell and/or taste disorders are significantly more frequent in cases of COVID-19 which is a common influenza and should be considered a focus for clinical suspicion and consideration to self-isolation procedures. Anosmia and ageusia are frequently early manifestation of COVID-19. Although pathogenetic mechanisms are still unclear, Vaira L. A. et al [12] proposed a potential explanation. Ageusia could be related to the highly expression of ACE2, identified as the cellular receptor for SARS-COV-2, on the oral mucosa and tongue. Anosmia seems not to be related to a mere local inflammatory condition in the nasal mucosa: in the absence of rhinitis symptoms, one hypothesis could be that the alterations are due to direct damage caused by the virus to the olfactory pathway.

Although many other neurological symptoms, such as headache, dizziness, conjunctivitis, and trigeminal neuralgia can be recorded in these patients, recent studies identified association with many neurological diseases. Guillain-Barre-Strohl syndrome (GBS) is an acute demyelinating polyradiculoneuropathy affecting cranial nerves, dorsal and ventral radices, spinal ganglia, and peripheral nerves. A type 4 hypersensitivity disorder is usually preceded by a respiratory (M. Pneumoniae, *H. influenzae*, CMV, EBV) or gastrointestinal (C. Jejuni). Sedaghat Z. et al [13], on 15 April 2020, described the first case of GBS apparently associated with COVID-19 infection. On the 24 April, another Italian case report [14] speculates the association between the acute polyradiculoneuropathy and COVID-19. The patient tested negative the most common abovementioned infections classically related to GBS. Although the study could not exclude the possibility of an autoimmune or paraneoplastic polyradiculoneuropathy mimicking GBS, the postinfectious etiology, the acute clinical course, and the typical neurophysiologic findings on electroneurography make alternative diagnosis less likely.

Poyiadji N. et al [15] found out that a woman with COVID-19 presenting with altered mental status had features on brain images consistent with acute necrotizing hemorrhagic encephalopathy (ANE). The authors concluded that the presence of the characteristic features of symmetric, multifocal lesions with thalamic involvement suggests that this is a case of acute necrotizing hemorrhagic encephalopathy associated with COVID-19. ANE is a rare complication of influenza and other viruses resulting from intracranial cytokine storm and resulting in blood-brain barrier damage, plausible with COVID-19 pathogenetic signature.

Although metabolic and electrolyte derangements, especially in thalamocortical pathways, secondary to COVID-19 are plausible causes of clinical or subclinical acute symptomatic seizures and status epilepticus [16], Lu L. et al [17] concluded that neither the virus nor potential risk factors for seizures increased the likelihood of acute symptomatic seizures in COVID-19.

### Clinical Case

**Fig. 3:**
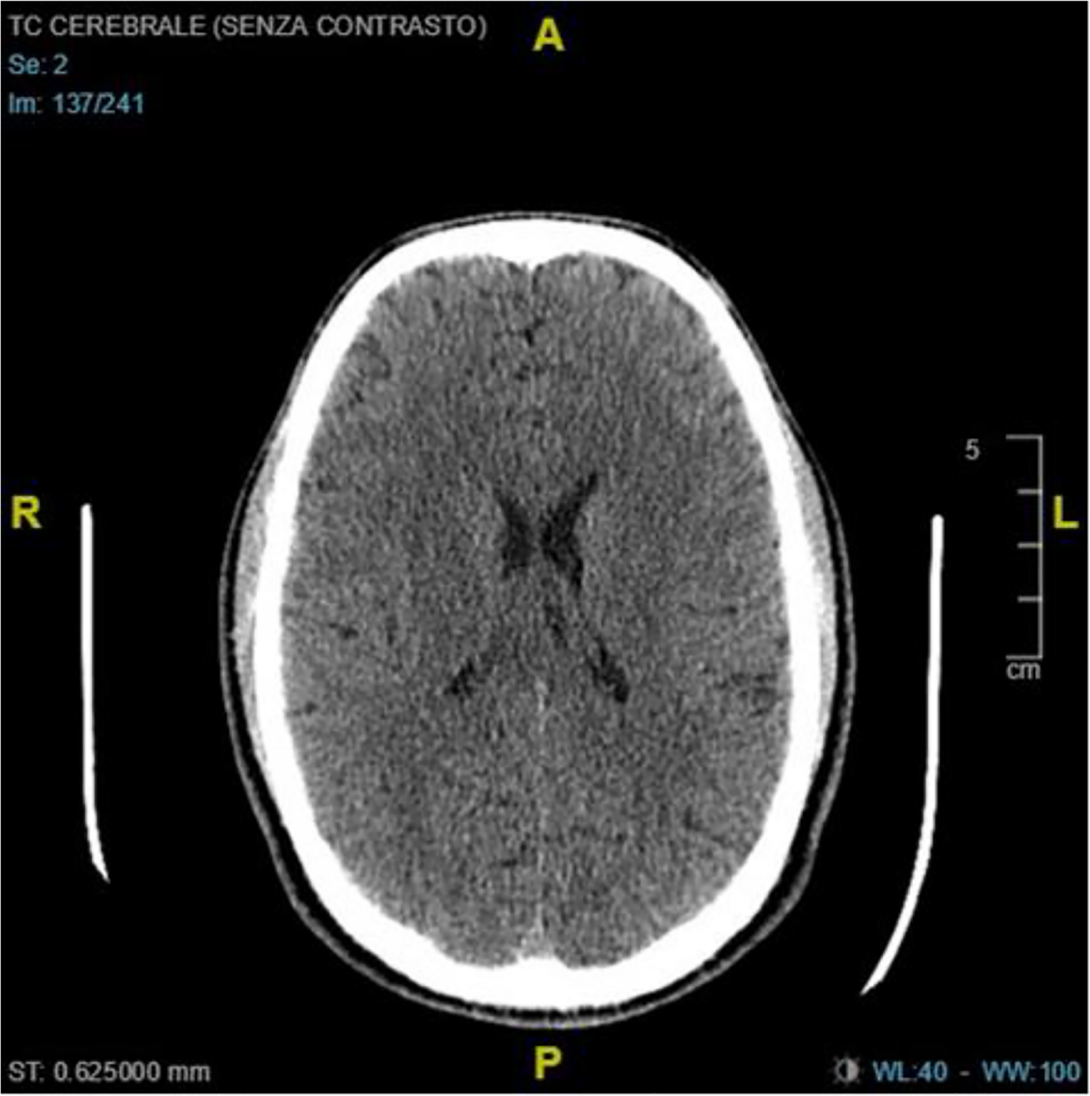
Normal brain CT scan at the entrance

**Fig. 4:**
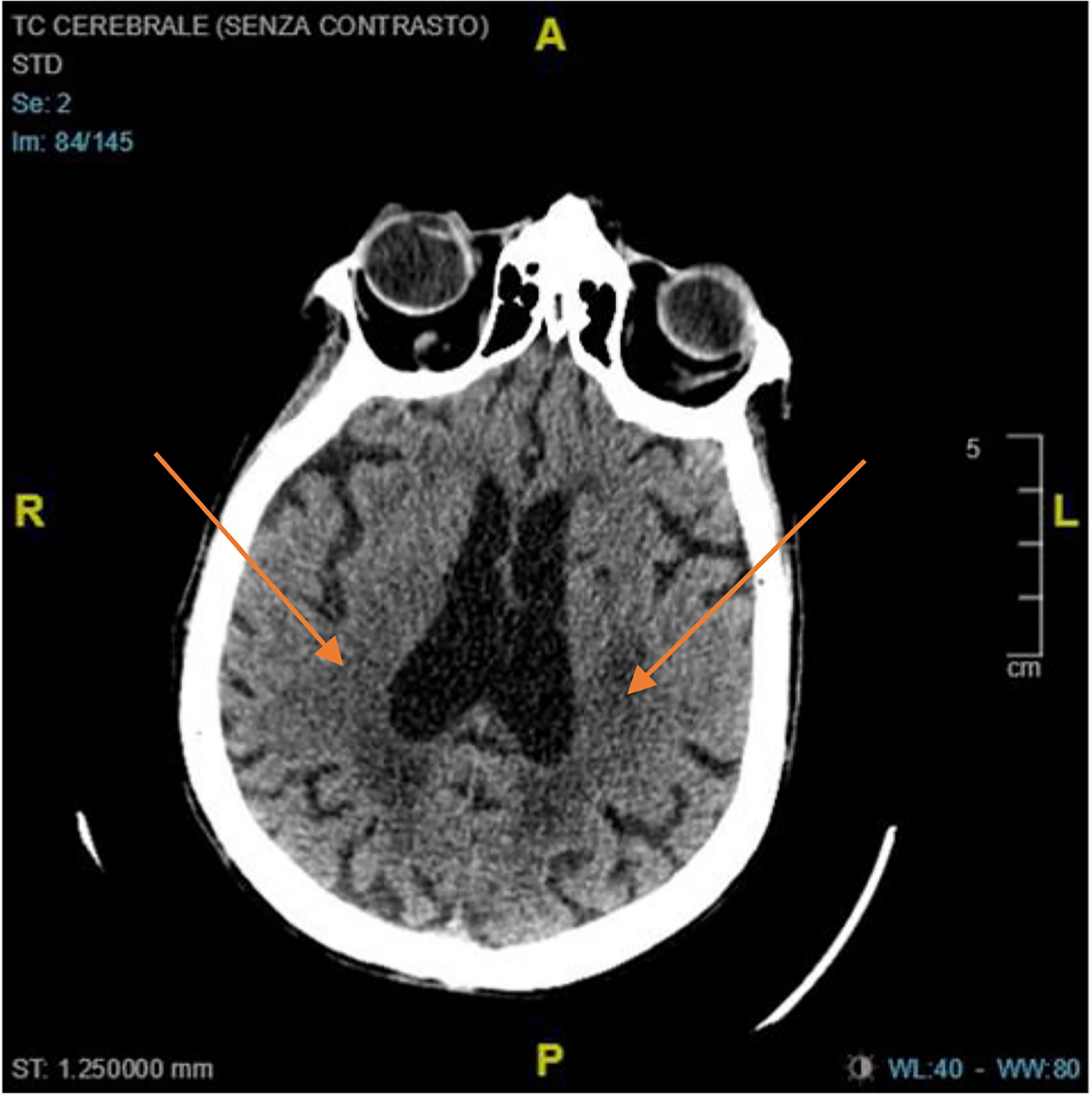
Ten days of brain CT with new periventricular ischemic lesions

A 72-year-old male arrived to emergency ward for altered brain status. SARS-COV-2 buffer was positive. Blood exams were quite normal. Chest CT scan showed interstitial pneumonia. Brain CT was normal at the entrance (Fig. 3). Patient was treated with antiviral therapy for 10 days. At discharge, another Brain CT showed new periventricular ischemic lesions (Fig. 4). Subject presented a mild cognitive impairment (Mini Mental State Examination was 23).

## Reproductive System

Little is still known about an association with reproductive system. ACE expression in testes poses patients at risk of orchitis. Pathologic analyses of testes of six patients who died of SARS revealed widespread germ cell destruction few or no spermatozoon in the seminiferous tubule, thickened basement membrane, and leukocyte infiltration. It further suggests that the reproductive functions should be followed and evaluated in recovered male SARS patients [18]**.**

## Conclusion

COVID-19 virus still represents a health and socioeconomic problem all over the world.

Clinicians must consider that it is no longer just a respiratory virus, as it has got a multi-organ trophism. It is important to consider extrapulmonary symptoms immediately to allow for early diagnosis and medical treatment.
